# Higher S-adenosylhomocysteine and lower ratio of S-adenosylmethionine to S-adenosylhomocysteine were more closely associated with increased risk of subclinical atherosclerosis than homocysteine

**DOI:** 10.3389/fnut.2022.918698

**Published:** 2022-08-10

**Authors:** Jinghe Xiao, Yiran You, Xu Chen, Yi Tang, Yuming Chen, Qiannan Liu, Zhaomin Liu, Wenhua Ling

**Affiliations:** ^1^Department of Nutrition, School of Public Health, Sun Yat-Sen University, Guangzhou, China; ^2^Guangdong Provincial Key Laboratory of Food, Nutrition, and Health, Guangzhou, China

**Keywords:** S-adenosylmethionine, S-adenosylhomocysteine, homocysteine, subclinical atherosclerosis, intima-media thickness

## Abstract

**Aim:**

To examine the relationship of C1 metabolites of the methionine cycle with the risk of subclinical atherosclerosis (SA) in the Chinese population.

**Methods:**

A total of 2,991 participants aged 45–75 years old were included for data analyses based on the baseline data of the Guangzhou Nutrition and Health Cohort. Three core serum methionine metabolites including serum S-adenosylmethionine (SAM), S-adenosylhomocysteine (SAH), and homocysteine (Hcy) were measured by UPLC-MS/MS. SA was determined by B-mode ultrasound measured carotid intima-media thickness (CIMT) at the common artery and bifurcation segments. Multivariable logistic and linear regression models were performed to estimate the associations of C1 metabolites of the methionine cycle with SA risk or CIMT.

**Results:**

After controlling for potential cofounders and other C1 metabolites, in comparison with the lowest quartile, participants in the highest quartile had lower risk of SA by 27.6% (OR = 0.724; 95% CI:0.563–0.93, *P*_trend_ = 0.007) for SAM and 32.2% (OR = 0.678; 95% CI:0.538–0.855, *P*_trend_ < 0.001) for SAM/SAH, while increased SA risk by 27.9% (OR = 1.279; 95% CI: 1.065–1.535, *P*_trend_ < 0.001) for SAH. No significant association was observed for Hcy with SA after further adjustment of SAH and SAM. The results of multivariable linear regression showed similar findings. The highest two standardized coefficients were observed for SAH (β = 0.104 for CCA and 0.121 for BIF, *P*< *0*.001) and SAM/SAH (β = −0.071 for CCA and −0.084 for BIF, *P*< *0*.001). Subgroup analyses suggested more evident associations of SAH with SA were observed in participants of higher cardiovascular risk profiles.

**Conclusion:**

Our cross-sectional data showed higher serum SAH, but lower SAM/SAH were independently associated with increased risk of SA among the Chinese middle-aged and elderly population.

## Introduction

Atherosclerosis is the underlying pathogenesis of the major cardiovascular disease (CVD) (1) inducing acute cardiovascular events or even death if plaque rupture and mural thrombosis occur at its advanced stage. Atherosclerosis has a long subclinical course without clinical manifestations. Subclinical atherosclerosis (SA) can be determined by ultrasound-measured carotid intima-media thickness (CIMT), a noninvasive measure and surrogate for CVD (2). A 10-year follow-up study (3) showed that people below the 25th percentile CIMT led reduced atherosclerotic CVD risk by 36–47%.

Epidemiological studies have established that hyper-homocysteinemia is an independent risk factor for atherosclerosis and contributes to plaque formation and development (4–6). However, large-scale interventional trials failed to observe cardiovascular benefits by lowering homocysteine (Hcy) levels *via* vitamin supplementation (7–9). The controversial findings might be due to dietary or supplemental folic acid, vitamin B6 or B12 could not alter the levels of S-adenosylhomocysteine (SAH) (10), the precursor of Hcy. Recent studies (11–13) suggested the toxicity of Hcy was secondary to SAH accumulation, and SAH might be a more sensitive biomarker or critical pathological factor of CVD than Hcy. SAH can be converted from S-adenosylmethionine (SAM), the primary methyl donor in the majority of biological methylation events, particularly DNA. Aberrant DNA methylation serves as an important mechanism prior to the onset of atherosclerosis (14). Hcy can be remethylated to SAM in an action that requires folate and vitamin B12. Hcy and its derivatives, SAH and SAM are thus referred to as C1 metabolites of the methylation cycle (15).

Our previous cohort study among Chinese patients undertaking coronary angiography has found plasma SAH independent of Hcy was positively associated with CVD events after 3 years of follow-up (13), and increased plasma SAM and SAM/SAH were independently associated with lowered overall and CVD mortality after 9.2 years of follow-up (16). Most of the previous human studies focused on SAH with CVD events or mortality. Nevertheless, observational studies on the subclinical course of atherosclerosis were limited and the relationship has never been reported in the Chinese population. Given the protracted subclinical phase and poor reversibility of atherosclerosis as well as the accident and fatal features of cardiovascular events, it is of critical importance in clinical practice to explore the associations of C1 metabolites with the risk of SA. The findings will promote early prediction and management of atherosclerosis and CVD events. We thus based on a Chinese cohort study to testify the associations of C1 metabolites of the methionine cycle with the risk of SA in the Chinese mid-life and elderly population.

## Methods

### Study population and baseline data collection

The research was a cross-sectional study based on the baseline data of the Guangzhou Nutrition & Health Cohort. A total of 3,169 participants aged 45–75 years who had lived in Guangzhou for above 5 years were recruited from October 2008 to June 2010 *via* multiple strategies (i.e., advertisements, health talks, referrals, etc.) in either communities or hospitals in Guangzhou, China. Participants with a medical history of diagnosed cancer, coronary heart disease (CHD), diabetes, renal failure, stroke, and Alzheimer's disease were excluded. The study protocol was approved by the Institute Ethics Committee of Sun Yat-sen University, and written informed consent was obtained from all participants before enrollment.

A structured questionnaire was administered *via* face-to-face interview by trained interviewers to collect baseline information on socio-demographic and lifestyle factors. Physical activity was evaluated using a validated 19-item physical activity questionnaire (17). A validated 79-item FFQ was used to estimate the habitual dietary intakes in the proceeding 1 year including total energy, macro-, and major micro-nutrients esp. folic acid, and vitamin B12 (18). Anthropometric measures were made for body weight, height, and waist circumference by standard methods, and body mass index (BMI) was calculated as weight in kilograms divided by the square of the height in meters. Blood pressure was measured two times on the left arm of the participants by standard methods, and the mean values were used for analyses.

### Blood collection and biochemical testing

Venous blood samples were collected after 8–12 h overnight fasting. Serum samples were separated after centrifugation at 4°C within 2 h after collection, then allocated into several vials and stored at −80°C until analyses. Serum total cholesterol (TC), total triglycerides (TG), low-density lipoprotein-cholesterol (LDL-c), high-density lipoprotein-cholesterol (HDL-c), and fasting blood glucose were measured. A detailed description of the biochemical methods and laboratory quality control indices has been published previously (19).

### C1 metabolites of the methionine cycle measurement

In this study, we detected three core serum methionine metabolites in the methionine cycle including SAM, SAH, and Hcy by HPLC–MS/MS methods (20–22) (Supplementary Table S1). Serum SAM/SAH was calculated as the methylation index.

Before testing, 10 μL of 50 mM DL-Dithiothreitol, and a 10 μL mixture of deuterium-labeled internal standards (^2^H_3_-SAM, 500 nM; ^2^H_4_-SAH, 500 nM; ^2^H_4_-Hcy, 5 μM) were added to the serum samples (50 μL) in turn. The mixtures were vortexed for 5 s and incubated at 37°C for 15 min in the dark. Then 30 μL perchloric acid (1 M) was added to samples for protein precipitation. Subsequently, the samples were centrifuged at 15,000 × g for 10 min at 4°C. Finally, the supernatants were filtered by a 0.22 μm membrane. The methionine metabolites were separated through an Acquity BEH C18 column (2.1 × 50 mm; i.d. 1.7 μm) (Waters Corp., Milford, MA, USA), detected by Agilent 1290 Infinity II UHPLC system coupled with Agilent 6410 Triple Quadrupole LC/MS system, and quantified in multiple reaction monitoring modes. The peaks and concentrations of the targets were measured by UPLC -MS/MS (Agilent) in the positive-ion (ESI+) mode (Supplementary Figure S1). The linearity regression coefficients of SAM, SAH, and Hcy were more than 0.99, with all the inter- and intra-assay coefficients of variation <10% (Supplementary Tables S2, S3).

### Carotid intima-media thickness (CIMT) measurement

The CIMT was measured bilaterally at the far wall of the artery using a high-resolution 7–12 MHz linear-array transducer system (Aplio; Toshiba, Japan) by a standardized scanning protocol (23). IMT was determined at two 10 mm segments: the distal segment of the common carotid artery (CCA) and bifurcation segment (BIF) (24). The wall thickness was measured under computer assistance using electronic calipers. B-mode images were recorded by two senior sonographers who were blinded to the identity of the participants and the study information. For the test-retest reliability, the correlation coefficients on the same day and ≥1 h apart were 0.979 and 0.982 for the same operator (*n* = 63), 0.853, and 0.89 between different operators (*n* = 22), respectively. In our analyses, CIMT was calculated as the mean value of bilaterals (19), and SA diagnosis was made if CIMT >0.93 mm (25) or focal IMT >2 mm (26).

### Statistical analyses

All the analyses were performed with SPSS 21 (IBM SPSS Inc, Chicago, IL). Statistical significance was inferred for a two-tailed *P*-value < 0.05. The baseline characteristics of participants were compared by the status of SA and shown in Table 1. Continuous data of normal distribution were expressed as mean ± *SD* and compared by student *t-*test. Variables of skewed distribution were presented as median (interquartile range) and compared by the Mann-Whitney U test. Categorical variables were compared by χ^2^ tests. Multivariable logistic regression was used to analyze the associations of quartiles of SAH, SAM, Hcy, and SAM/SAH (Table 2) as well as dietary intakes of folic acid and vitamin B12 (Table 3) with the risk of SA after adjustment of a range of potential confounders in different models (detailed covariates and the structured models were shown in the footnotes of Tables). Multivariable linear regression (Table 4) was used to testify the associations between serum C1 metabolites (SAM, SAH, Hcy, and SAM/SAH) with CIMT (CCA and BIF). Owing to the metabolic interplay among SAM, SAH, and Hcy, we conducted further sensitivity analyses to testify to the independent association of individual C1 metabolites with SA risk or CIMT levels by the mutual adjustment of the other two metabolites in the logistic or linear regression models (shown in Model 4 of Tables 2, 3).

**Table 1:** Baseline **c**haracteristics and selected risk factors of the study participants by presence or not of subclinical atherosclerosis, Guangzhou Nutrition and Health Cohort (*n* = 2,991)^a^.

**Characteristic**	**Subclinical atherosclerosis**		**Total (*n =* 2991)**	***P***
	**No (*n =* 1,533)**	**Yes (*n =* 1,458)**		
Male (%)	357 (23.3)	588 (40.3)	945 (31.6)	<0.001
Age (years)	59.0 ± 5.3	62.3 ± 5.9	60.6 ± 5.8	<0.001
BMI (kg/m^2^)	23.1 ± 3.1	24.1 ± 3.1	23.6 ± 3.2	<0.001
Waist circumference (cm)	83.5 ± 8.7	86.4 ± 8.6	84.9 ± 8.8	<0.001
**Education (years)**				0.004
≤6	120 (7.8)	128 (8.8)	248 (8.3)	
7–12	1,061 (69.2)	927 (63.6)	1,988 (66.5)	
>12	352 (23.0)	403 (27.6)	755 (25.2)	
**Monthly income (Yuan/person)**				0.373
<1,500	113 (7.4)	104 (7.2)	217 (7.3)	
1,500–3,000	764 (49.8)	693 (47.5)	1,457 (48.7)	
>3,000	656 (42.8)	661 (45.3)	1,317 (44.0)	
Physical activity (MET·h/d)	34.2 ± 5.7	33.8 ± 5.6	34.0 ± 5.6	0.034
Smoker (%)^b^	130 (8.5)	120 (8.2)	250 (8.4)	0.805
Alcohol drinker (%)^c^	103 (6.7)	133 (9.1)	236 (7.9)	0.015
Tea drinker (%)^d^	809 (52.8)	877 (60.2)	1686 (56.4)	<0.001
Systolic blood pressure (mmHg)	121 ± 17	131 ± 18	126 ± 18	<0.001
Diastolic blood pressure (mmHg)	74 ± 10	77 ± 10	76 ± 10	<0.001
**Dietary intakes**				
Total energy (kcal/d)	1584.3 ± 513.7	1623.8 ± 507.9	1603.6 ± 511.2	0.035
Total fat (g/d)	51.3 ± 21.2	53.5 ± 22.4	52.3 ± 21.8	0.006
Total protein (g/d)	66.5 ± 23.2	67.3 ± 22.4	66.9 ± 22.8	0.328
Folic acid (μg/d)	210.4 ± 89.5	210.3 ±77.5	210.4 ± 83.8	0.986
Vitamin B12 (μg/d)	1.4 ± 1.1	1.4 ± 0.9	1.4 ± 1.0	0.698
**Biochemical testing**				
Total cholesterol (mmol/l)	5.59 ± 1.03	5.57 ± 1.07	5.58 ± 1.05	0.054
LDL-c (mmol/l)	3.52 ± 0.90	3.68 ± 0.92	3.63 ± 0.91	0.025
HDL-c (mmol/l)	1.49 ± 0.42	1.37 ± 0.39	1.43 ± 0.41	<0.001
Total triglyceride (mmol/l)	1.52 ± 1.28	1.55 ±1.09	1.54 ± 1.19	0.491
Fasting glucose (mmol/l)	4.92 ± 1.15	5.11 ± 1.26	5.01 ± 1.21	<0.001
Homocysteine (μmol/l)	12.6 (11.1–15.2)	13.7 (11.6–17.4)	13.1 (11.3–16.4)	<0.001
SAM (nmol/l)	86.9 (74.7–107.6)	90.9 (75.6–113.1)	88.2 (75.0–110.4)	0.001
SAH (nmol/l)	14.6 (11.0–22.7)	18.0 (12.0–26.1)	15.8 (11.5–24.5)	<0.001
SAM/SAH	6.2 (4.4–7.5)	5.3 (4.1–6.9)	5.7 (4.2–7.1)	<0.001
**Carotid-IMT**				
CCA (mm)	0.84 ± 0.07	1.02 ± 0.11	0.93 ± 0.13	<0.001
BIF (mm)	0.93 ± 0.13	1.07± 0.19	1.00 ± 0.18	<0.001

a *Continuous data of normal distribution were expressed as mean ± standard deviation and compared by t test. Continuous variables of skewed distribution were presented as median (interquartile range) and compared by the Wilcoxon's rank sum test. Categorical variables were compared by χ^2^ test. The carotid IMT >0.93mm or focal IMT >2 mm was defined as subclinical atherosclerosis*.

b *Smoker, ≥1 cigarette/d in the past year*.

c *Alcohol drinker, ≥1 cup/wk in the past year*.

d *Tea drinker, ≥1 cup/wk in the past year*.

*BIF: Bifurcation segment; BMI: Body mass index; CCA: Common carotid artery segment; HDL-c: High-density lipoprotein-cholesterol; IMT: Intima-media thickness; LDL-c: Low-density lipoprotein-cholesterol; MET: Metabolic equivalent; SAH: S-adenosylhomocysteine; SAM: S-adenosylmethionine*.

**Table 2:** ORs (95% CIs) for the occurrence of subclinical atherosclerosis by quartiles of SAM, SAH, homocysteine and SAM/SAH levels, Guangzhou Nutrition and Health Cohort (*n* = 2991)^a^.

**ORs (95%CIs) of subclinical atherosclerosis**	**Quartiles of C1 metabolites of the methionine cycle**				***P*** **_trend_**	**Each 1-SD increase^b^**
	**Q1 (*n =* 747)**	**Q2 (*n =* 748)**	**Q3 (*n =* 748)**	**Q4 (*n =* 748)**		
**SAM (nmol/l)**						
Median (minimum–maximum)	68.0 (22.2–75.0)	80.9 (75.1–88.2)	97.7 (88.3–110.4)	124.5 (110.5–228.5)		
Model 1 (age and gender adjusted OR)	1.000	0.912 (0.737, 1.129)	0.956 (0.771, 1.184)	1.103 (0.889, 1.369)	0.329	1.051 (0.971,1.139)
Model 2^c^	1.000	0.854 (0.660, 1.029)	0.864 (0.690, 1.080)	0.944 (0.752, 1.185)	0.740	0.990 (0.910, 1.077)
Model 3^d^	1.000	0.853 (0.659, 1.028)	0.868 (0.694, 1.086)	0.947 (0.755, 1.189)	0.772	0.991 (0.911, 1.078)
Model 4^e^	1.000	0.802 (0.641, 1.003)	0.697 (0.549, 0.886)	0.724 (0.563, 0.930)	0.007	0.896 (0.818, 0.982)
**SAH (nmol/l)**						
Median (minimum–maximum)	10.0 (5.6–11.5)	13.1 (11.6–15.8)	20.3 (15.9–24.5)	28.7 (24.6–44.5)		
Model 1 (age and gender adjusted OR)	1.000	1.006 (0.805, 1.317)	1.375 (1.170, 1.794)	1.667 (1.416, 1.931)	<0.001	1.362 (1.165, 1.563)
Model 2^c^	1.000	0.990 (0.791, 1.239)	1.292 (1.024, 1.506)	1.431 (1.309, 1.813)	<0.001	1.293 (1.103, 1.497)
Model 3^d^	1.000	0.991 (0.705, 1.208)	1.256 (1.018, 1.498)	1.360 (1.258, 1.772)	<0.001	1.271 (1.035, 1.408)
Model 4^e^	1.000	0.851 (0.681, 1.133)	1.113 (1.007, 1.376)	1.279 (1.065, 1.535)	<0.001	1.210 (1.017, 1.342)
**Homocysteine (μmol/)**						
Median (minimum–maximum)	10.3 (3.8–11.3)	12.1 (11.4–13.1)	14.3 (13.2–16.4)	19.2 (16.5–58.3)		
Model 1 (age and gender adjusted OR)	1.000	1.062 (0.855, 1.318)	1.410 (1.137, 1.749)	1.744 (1.404, 2.166)	<0.001	1.256 (1.159, 1.356)
Model 2^c^	1.000	0.982 (0.785, 1.230)	1.264 (1.009, 1.583)	1.482 (1.180, 1.861)	<0.001	1.178 (1.091, 1.274)
Model 3^d^	1.000	0.979 (0.782, 1.225)	1.257 (1.003, 1.576)	1.474 (1.174, 1.852)	<0.001	1.176 (1.083, 1.273)
Model 4^e^	1.000	0.939 (0.749, 1.177)	1.135 (0.916, 1.455)	1.246 (0.975, 1.593)	0.029	1.116 (1.020, 1.217)
**SAM/SAH**						
Median (minimum–maximum)	3.6 (1.2–4.2)	5.0 (4.3–5.7)	6.5 (5.8–7.2)	8.1 (7.3–18.0)		
Model 1 (age and gender adjusted OR)	1.000	1.086 (0.876, 1.346)	0.782 (0.631, 0.969)	0.612 (0.493, 0.759)	<0.001	0.798 (0.735, 0.860)
Model 2^c^	1.000	1.143 (0.915, 1.428)	0.847 (0.678, 1.058)	0.629 (0.502, 0.787)	<0.001	0.813 (0.755, 0.887)
Model 3^d^	1.000	1.144 (0.916, 1.429)	0.846 (0.677, 1.057)	0.629 (0.502, 0.787)	<0.001	0.816 (0.757, 0.890)
Model 4^e^	1.000	1.177 (0.941, 1.472)	0.889 (0.709, 1.114)	0.678 (0.538, 0.855)	<0.001	0.869 (0.771, 0.914)

a *ORs and 95% CIs were estimated by multivariable logistic regression models with covariates being adjusted by enter methods. The carotid IMT >0.93 mm or focal IMT >2 mm was defined as subclinical atherosclerosis*.

b *The metabolites of SAM, SAH and homocysteine concentrations were natural logarithm transformed, standardized and treated as continuous variables in the multivariable logistic regression models*.

c *The adjusted covariates in Model 2 included age (years), sex (men or women), BMI (kg/m^2^), waist circumference (cm), education (≤6, 7-12, or >12 years), monthly income (<1500, 1500–3000, or >3000 yuan), physical activity (MET·h/d), smoking status (yes or no), alcohol drinking (yes or no), tea drinking (yes or no), systolic blood pressure (mmHg), energy intake (kcal/d), fat intake (g/d), protein intake(g/d), LDL-c (mmol/l), HDL-c (mmol/l), total triglyceride (mmol/l), and fasting glucose (mmol/l)*.

d *Model 3 was adjusted for the covariates in model 2 plus dietary folic acid and vitamin B12 intakes (μg/d)*.

e *Model 4 was a sensitivity analysis to testify the independent association of individual C1 metabolite due to the interplay of SAM, SAH and Hcy. The covariates of Model 4 was based on Model 3 additionally adjusted for SAH (nmol/l) and homocysteine (μmol/l) if SAM (nmol/l) was the independent variable; SAM (nmol/l) and homocysteine (μmol/l) if SAH (nmol/l) was the independent variable; SAM (nmol/l) and SAH (nmol/l) if homocysteine (μmol/l) was the independent variable; or homocysteine (μmol/l) for SAM/SAH as the independent variable*.

*BMI: Body mass index; HDL-c: High-density lipoprotein-cholesterol; IMT: Intima-media thickness; LDL-c: Low-density lipoprotein-cholesterol; MET: Metabolic equivalent; SAH: S-adenosylhomocysteine; SAM: S-adenosylmethionine*.

**Table 3:** ORs (95% CIs) for the prevalence of subclinical atherosclerosis according to quartiles of dietary folic acid and vitamin B12 intakes, Guangzhou Nutrition and Health Cohort (*n* = 2,991)^a^.

**ORs (95%CIs) of subclinical atherosclerosis**	**Quartile**				***P*** **_trend_**	**Each 1-SD increase**
	**1**	**2**	**3**	**4**		
**Folic acid (μg/d)**						
*n*	748	748	748	747		
Median (minimum–maximum)	136.2 (44.6–157.7)	177.1 (157.8–197.4)	219.8 (197.5–245.9)	290.7 (246.0–336.4)		
Model 1 (age and gender adjusted OR)	1.000	1.117 (0.902, 1.384)	1.056 (0.852, 1.310)	1.129 (0.910, 1.401)	0.375	1.027 (0.951, 1.108)
Model 2^b^	1.000	1.087 (0.868, 1.362)	1.054 (0.831, 1.336)	1.031 (0.803, 1.324)	0.887	0.994 (0.907, 1.088)
Model 3^c^	1.000	1.077 (0.859, 1.350)	1.053 (0.829, 1.337)	1.029 (0.800, 1.324)	0.880	0.998 (0.910, 1.093)
Model 4^d^	1.000	1.106 (0.879, 1.391)	1.117 (0.866, 1.441)	1.163 (0.855, 1.581)	0.347	1.049 (0.932, 1.181)
**Vitamin B12 (μg/d)**						
*n*	748	748	747	748		
Median (minimum–maximum)	0.6 (0.1–0.8)	1.0 (0.9–1.2)	1.4 (1.3–1.7)	2.3 (1.8–2.9)		
Model 1 (age and gender adjusted OR)	1.000	1.164 (0.939, 1.443)	1.085 (0.875, 1.346)	1.179 (0.951, 1.461)	0.221	1.003 (0.930, 1.081)
Model 2^b^	1.000	1.166 (0.926, 1.467)	1.087 (0.857, 1.377)	1.096 (0.851, 1.412)	0.620	0.945 (0.861, 1.037)
Model 3^c^	1.000	1.181 (0.937, 1.489)	1.079 (0.850, 1.370)	1.105 (0.857, 1.427)	0.618	0.948 (0.862, 1.041)
Model 4^d^	1.000	1.185 (0.940, 1.495)	1.084 (0.853, 1.378)	1.115 (0.861, 1.443)	0.582	(0.863, 1.042)

a *ORs and 95% CIs were estimated by multivariable logistic regression models with covariates being adjusted by enter methods. The carotid IMT >0.93 mm or focal IMT >2 mm was defined as subclinical atherosclerosis. Dietary intake of folic acid and vitamin B12 were adjusted by total energy intake using residual method*.

b *The adjusted covariates in Model 2 included age (years), sex (men or women), BMI (kg/m^2^), waist circumference (cm), education (≤6, 7–12, or >12 years), monthly income (<1,500, 1,500–3,000, or >3,000 yuan), physical activity (MET·h/d), smoking status (yes or no), alcohol drinking (yes or no), tea drinking (yes or no), systolic blood pressure (mmHg), energy intake (kcal/d), fat intake (g/d), protein intake(g/d), LDL-c (mmol/l), HDL-c (mmol/l), total triglyceride (mmol/l), and fasting glucose (mmol/l)*.

c *Model 3 was adjusted for the covariates in model 2 plus concentrations of serum SAM (nmol/l), SAH (nmol/l) and homocysteine (μmol/l)*.

d *Model 4 was based on the covariates in Model 3 additionally adjusted for dietary vitamin B12 intake (μg/d) if folic acid intake (μg/d) was the independent variable, or dietary folic acid intake (μg/d) if vitamin B12 intake (μg/d) was the independent variable*.

*BMI, Body mass index; HDL-c, High-density lipoprotein-cholesterol; IMT, Intima-media thickness; LDL-c, Low-density lipoprotein-cholesterol; MET, Metabolic equivalent; SAH, S-adenosylhomocysteine; SAM, S-adenosylmethionine*.

**Table 4:** Multivariable linear regression on the associations of C1 metabolites of the methionine cycle and carotid intima-media thickness, Guangzhou Nutrition and Health Cohort (*n* = 2,991)^a^.

**ORs (95%CIs)**	**SAM (nmol/l)**			**SAH (nmol/l)**			**Homocysteine (μmol/l)**			**SAM/SAH**		
	**B (SE)**	**β**	***P***	**B (SE)**	**β**	***P***	**B (SE)**	**β**	***P***	**B (SE)**	**β**	***P***
**CIMT-CCA (μm)**												
Model 1^b^	0.512 (0.087)	0.100	<0.001	2.639 (0.270)	0.165	<0.001	3.757 (0.500)	0.127	<0.001	−6.742 (1.020)	−0.112	<0.001
Model 2^c^	0.364 (0.086)	0.071	<0.001	2.088 (0.266)	0.130	<0.001	2.755 (0.489)	0.093	<0.001	−5.485 (0.991)	−0.091	<0.001
Model 3^d^	0.365 (0.086)	0.071	<0.001	2.087 (0.266)	0.130	<0.001	2.749 (0.489)	0.093	<0.001	−5.475 (0.991)	−0.091	<0.001
Model 4^e^	0.080 (0.095)	0.016	0.401	1.665 (0.315)	0.104	<0.001	1.627 (0.518)	0.055	0.002	−4.317 (1.024)	−0.071	<0.001
**CIMT-BIF (μm)**												
Model 1^b^	0.225 (0.126)	0.031	0.075	2.895 (0.391)	0.128	<0.001	3.117 (0.725)	0.075	<0.001	−9.073 (1.470)	−0.106	<0.001
Model 2^c^	0.089 (0.127)	0.012	0.483	2.407 (0.395)	0.106	<0.001	2.160 (0.724)	0.052	0.003	−7.849 (1.464)	−0.092	<0.001
Model 3^d^	0.085 (0.127)	0.012	0.502	2.399 (0.395)	0.106	<0.001	2.177 (0.725)	0.052	0.003	−7.842 (1.465)	−0.092	<0.001
Model 4^e^	−0.332 (0.141)	−0.046	0.019	2.735 (0.467)	0.121	<0.001	0.730 (0.769)	0.017	0.342	−7.180 (1.517)	−0.084	<0.001

a *B(SE) and β were estimated by multivariable linear regression with covariates being adjusted by enter methods*.

b *The adjusted covariates in Model 1 included age (years) and sex (men or women)*.

c *Model 2 included age (years), sex (men or women), BMI (kg/m^2^), waist circumference (cm), education (≤6, 7–12, or >12 years), monthly income (<1,500, 1,500–3,000, or >3,000 yuan), physical activity (MET·h/d), smoking status (yes or no), alcohol drinking (yes or no), tea drinking (yes or no), systolic blood pressure (mmHg), energy intake (kcal/d), fat intake (g/d), protein intake(g/d), LDL-c (mmol/l), HDL-c (mmol/l), total triglyceride (mmol/l), and fasting glucose (mmol/l)*.

d *Model 3 was adjusted for the covariates in model 2 plus dietary folic acid and vitamin B12 intakes (μg/d)*.

e *Model 4 was a sensitivity analysis to testify the independent association of individual C1 metabolite with the carotid IMT (CCA and BIF). The covariates in Model 4 were based on the covariates in Model 3 additionally adjusted for SAH (nmol/l) and homocysteine (μmol/l) if SAM (nmol/l) was the independent variable; SAM (nmol/l) and homocysteine (μmol/l) if SAH (nmol/l) was the independent variable; SAM (nmol/l) and SAH (nmol/l) if homocysteine (μmol/l) was the independent variable; or homocysteine (μmol/l) if SAM/SAH was the independent variable*.

*B, Unstandardized coefficient. SE, Standard error. β, Standardized coefficient. BIF, Bifurcation segment; BMI, Body mass index; CCA, Common carotid artery segment; CIMT, carotid intima-media thickness; HDL-c, High-density lipoprotein-cholesterol; LDL-c, Low-density lipoprotein-cholesterol; MET, Metabolic equivalent; SAH, S-adenosylhomocysteine; SAM, S-adenosylmethionine*.

Multiplicative interactions (Supplementary Table S4) were examined before subgroup analyses by including the product terms of quartiles of the C1 metabolites with the stratified variables (age, sex, BMI, blood pressure, alcohol drinking, intakes of folic acid and vitamin B12, LDL-c, HDL-c, total triglyceride, and fasting glucose as well as Hcy) in the logistic models. Subgroup analyses were subsequently performed by multivariable logistic regression for the stratified variables if their *P*-values for interactions were <0.15.

## Results

### Characteristics of the study participants

The baseline characteristics of the participants are shown in Table 1. A total of 2,991 men and women (*n* = 2,046, 68.4%) were included in the analyses after exclusion of any missing value of SAM, SAH, or Hcy. The prevalence of SA in this study was 48.7% of which 40.3% of cases were men. In comparison with those of non-SA, participants of SA were more likely to be older, had higher BMI, waist circumference, systolic and diastolic blood pressure, higher rates of habitual alcohol and tea drinking, higher dietary intakes of energy and total fat, higher levels of serum LDL-c, fasting glucose, serum SAM, SAH, and Hcy, but lower levels of total physical activity, HDL-c, and SAM/SAH. Obvious inter-correlations were observed between these C1 metabolites, with Spearman correlation coefficients being 0.529 for SAM and SAH, 0.192 for SAM and Hcy, and 0.413 for SAH and Hcy (data not shown).

### Associations between serum C1 metabolites of the methionine cycle as well as dietary folic acid and vitamin B12 with SA risk

The results of multivariable logistic regression (Table 2) indicated that after controlling for potential confounders (Model 3), compared with the lowest quartile group, participants in highest quartile had significantly increased risk of SA by 36% (OR = 1.36, 95% CI: 1.258–1.772, *P*_trend_ < 0.001) for SAH and 47.4% (OR = 1.474; 95% CI: 1.174–1.852, *P*_trend_ < 0.001) for Hcy, while decreased SA risk for SAM/SAH by 37.1% (OR = 0.629, 95% CI:0.502–0.787, *P*_trend_ < 0.001). No significant association was observed between SAM and SA risk in Model 3.

Sensitivity analyses by the mutual adjustment of the other methionine metabolites (Model 4) indicated obvious attenuation of ORs for SAM, SAH, or Hcy. In contrast, further adjustment of SAH and Hcy made the originally null association of SAM with SA in model 3 became statistically significant in model 4 and resulted in a lowered SA risk by 27.6% (OR = 0.724; 95% CI:0.563–0.93, *P*_trend_ = 0.007) by comparison of the two extreme quartiles. The association of Hcy and SA was attenuated to non-significance (OR = 1.246, 95% CI:0.975–1.593) by comparison of the two extreme quartiles although a notable dose-response relationship remained (*P*_trend_ = 0.029) after additional adjustment of SAM and SAH. Additionally, further adjustment of Hcy, the OR of SAM/SAH increased from 0.629 (95% CI:0.502–0.787, *P*_trend_ < 0.001**)** to 0.678 (95% CI:0.538–0.855, *P*_trend_ < 0.001) by comparison of the two extreme quartiles. One SD increase of natural-logarithm transformed metabolites were associated with decreased SA risk by 10.4% (OR = 0.896; 95% CI:0.818–0.982) for SAM, and 13.1% (OR = 0.869; 95% CI:0.771–0.914) for SAM/SAH, while increased SA risk by 21% (OR = 1.21; 95% CI: 1.017–1.342) for SAH and 11.6% (OR = 1.116; 95% CI: 1.02–1.217) for Hcy. No significant associations were observed between dietary folic acid and vitamin B12 intakes with SA risk (Table 3).

### Associations of serum C1 metabolites of the methionine cycle with CIMT

After controlling potential confounders, the results of multivariable linear regression (Model 4) indicated that SAH and Hcy were positive while SAM and SAM/SAH were negatively associated with CIMT, with the first two largest standardized coefficients being observed for SAH (β = 0.121, *P*< *0*.001) and SAM/SAH (β = −0.084, *P*< *0*.001) with CIMT-BIF levels (Table 4). With each 1 nmol/l increase of SAH, CIMT-CCA and CIMT-BIF increased by 1.665 and 2.735 μm, respectively. SAM had significant and inverse association with CIMT-BIF only (B = −0.332, *P* = 0.019). Despite inter-correlation, SAM/SAH was more closely related to CIMT than other C1 metabolites of the methionine cycle. Each unit increase of SAM/SAH was associated with decreased CIMT-CCA and BIF by 4.317 and 7.180 μm, respectively, while per 1 μmol/L increase of Hcy was associated with increased CIMT-CCA by 1.627 μm.

### Subgroup analyses

Subgroup analyses (Table 5) were only conducted for variables of *P* for interaction <0.15. The subgroup results suggested more evident associations being observed for SAH and SA risk among habitual alcohol drinkers (OR = 3.762; 95% CI: 1.327–10.669, *P*_trend_ = 0.012), participants of higher levels of LDL-c (OR = 1.649; 95%CI:1.225–2.220, *P*_trend_ = 0.001), fasting glucose (OR = 3.268; 95% CI:1.421–7.515, *P*_trend_ = 0.004) and Hcy (OR=2.351; 95% CI:1.590–3.477, *P*_trend_ < 0.001). For Hcy, stronger associations with SA risk were observed in participants elder than 65y (OR = 2.508; 95% CI: 1.453–4.330, *P*_trend_ < 0.001), habitual alcohol drinkers (OR = 2.097; 95% CI: 1.871–5.049, *P*_trend_ = 0.023), and of lower HDL-c levels (OR = 2.225; 95% CI: 1.159–4.271, *P*_trend_ = 0.037). For SAM/SAH, reduced SA risk as more evident in participants of habitual alcohol drinking (OR = 0.331; 95% CI:0.131–0.84, *P*_trend_ = 0.013), of lower LDL-c levels (OR = 0.515; 95% CI:0.374–0.708, *P*_trend_ < 0.001), with lower fasting glucose levels (OR = 0.293; 95% CI:0.13–0.665, *P*_trend_ < 0.001) and with lower Hcy (OR = 0.459; 95% CI:0.319–0.66, *P*_trend_ < 0.001).

**Table 5:** Subgroup analyses by age, gender and selected cardiovascular risk factors on the risk of subclinical atherosclerosis according to the quartiles of serum C1 metabolites of the methionine cycle, Guangzhou Nutrition and Health Cohort (*n* = 2,991)^a^.

**ORs (95%CIs) of subclinical atherosclerosis**	**Cases/*n***	**ORs (95% CIs) of Q4 vs Q1**	***P*** **_trend_**	***P*** **for interaction**
**SAM (nmol/l)**				
**Age (years)**				0.548
<65	1,007/2,337	0.995 (0.775, 1.279)	0.897	
≥65	451/654	1.106 (0.647, 1.890)	0.509	
**Sex**				0.156
Female	870/2,046	1.096 (0.835, 1.439)	0.482	
Male	588/945	0.702 (0.459, 1.074)	0.156	
**Alcohol drinking**				0.304
No	1,325/2,755	0.937 (0.741, 1.186)	0.662	
Yes	133/236	1.170 (0.438, 3.126)	0.524	
**LDL-c (mmol/l)**				0.069
<3.4	594/1,262	0.766 (0.536, 1.095)	0.145	
≥3.4	864/1,729	1.104 (0.820, 1.486)	0.394	
**HDL-c (mmol/l)**				0.090
<1.0	222/381	0.584 (0.293, 1.164)	0.128	
≥1.0	1,236/2,610	1.000 (0.784, 1.276)	0.756	
**Fasting glucose (mmol/l)**				0.397
<6.1	1,280/2,708	0.987 (0.776, 1.254)	0.896	
≥6.1	178/283	0.641 (0.279, 1.472)	0.591	
**Homocysteine (μmol/l)**				0.204
<13.1	630/1,510	0.784 (0.572, 1.075)	0.131	
≥13.1	828/1,481	0.986 (0.696, 1.398)	0.937	
**SAH (nmol/l)**				
**Age (years)**				0.883
<65	1,007/2,337	1.570 (1.221, 2.018)	<0.001	
≥65	451/654	1.865 (1.081, 3.219)	0.028	
**Sex**				0.639
Female	870/2,046	1.505 (1.145, 1.979)	0.001	
Male	588/945	1.597 (1.048, 2.432)	0.016	
**Alcohol drinking**				0.109
No	1,325/2,755	1.468 (1.159, 1.859)	<0.001	
Yes	133/236	3.762 (1.327, 10.669)	0.012	
**LDL-c (mmol/l)**				0.717
<3.4	594/1,262	1.393 (1.073, 1.996)	0.012	
≥3.4	864/1,729	1.649 (1.225, 2.220)	0.001	
**HDL-c (mmol/l)**				0.737
<1.0	222/381	1.906 (0.966, 3.761)	0.091	
≥1.0	1,236/2,610	1.511 (0.983, 1.931)	0.132	
**Fasting glucose(mmol/l)**				0.151
<6.1	1,280/2,708	1.417 (1.114, 1.801)	0.001	
≥6.1	178/283	3.268 (1.421, 7.515)	0.004	
**Homocysteine (μmol/l)**				
<13.1	630/1,510	0.946 (0.650, 1.377)	0.773	0.001
≥13.1	828/1,481	2.351 (1.590, 3.477)	<0.001	
**Homocysteine (μmol/l)**				
Age (years)				0.020
<65	1,007/2,337	1.383 (1.076, 1.778)	0.003	
≥65	451/654	2.508 (1.453, 4.330)	<0.001	
**Sex**				0.163
Female	870/2,046	1.348 (1.020, 1.782)	0.013	
Male	588/945	1.853 (1.232, 2.785)	0.001	
**Alcohol drinking**				0.149
No	1,325/2,755	1.446 (1.140, 1.834)	0.001	
Yes	133/236	2.097 (1.871, 5.049)	0.023	
**LDL-c (mmol/l)**				0.334
<3.4	594/1,262	1.368 (0.959, 1.951)	0.065	
≥3.4	864/1,729	1.549 (0.847, 2.091)	0.079	
**HDL-c (mmol/l)**				0.593
<1.0	222/381	2.225 (1.159, 4.271)	0.037	
≥1.0	1,236/2,610	1.408 (1.102, 1.800)	0.001	
**Fasting glucose(mmol/l)**				0.819
<6.1	1,280/2,708	1.459 (1.147, 1.857)	<0.001	
≥6.1	178/283	1.884 (1.064, 4.108)	0.043	
**SAM/SAH**				
**Age (years)**				0.874
<65	1,007/2,337	0.611 (0.478, 0.782)	<0.001	
≥65	451/654	0.619 (0.365, 0.948)	0.044	
**Sex**				0.134
Female	870/2,046	0.689 (0.526, 0.904)	0.002	
Male	588/945	0.527 (0.350, 0.793)	<0.001	
**Alcohol drinking**				0.239
No	1,325/2,755	0.651 (0.515, 0.822)	<0.001	
Yes	133/236	0.331 (0.131, 0.840)	0.013	
**LDL-c (mmol/l)**				0.046
<3.4	594/1,262	0.515 (0.374, 0.708)	<0.001	
≥3.4	864/1,729	0.779 (0.960, 1.171)	0.029	
**HDL-c (mmol/l)**				0.778
<1.0	222/381	0.595 (0.311, 1.136)	0.058	
≥1.0	1,236/2,610	0.628 (0.493, 1.799)	0.097	
**Fasting glucose(mmol/l)**				0.016
<6.1	1,280/2,708	0.293 (0.130, 0.665)	<0.001	
≥6.1	178/283	0.672 (0.531, 0.851)	<0.001	
**Homocysteine (μmol/l)**				0.010
<13.1	630/1,510	0.459 (0.319, 0.660)	<0.001	
≥13.1	828/1,481	1.010 (0.725, 1.407)	0.955	

a *ORs and 95% CIs were estimated by multivariable logistic regression models with covariates being adjusted by enter methods. All of the regression models were adjusted for age (years), sex (men or women), BMI (kg/m^2^), waist circumference (cm), education (≤6, 7–12, or >12 years), monthly income (<1,500, 1,500–3,000, or >3,000 yuan), physical activity (MET·h/d), smoking status (yes or no), alcohol drinking (yes or no), tea drinking (yes or no), systolic blood pressure (mmHg), energy intake (kcal/d), fat intake (g/d), protein intake(g/d), dietary folic acid and vitamin B12 intake (μg/d), LDL-c (mmol/l), HDL-c (mmol/l), total triglyceride (mmol/l), and fasting glucose (mmol/l)*.

*BMI, Body mass index; HDL-c, High-density lipoprotein-cholesterol; LDL-c, Low-density lipoprotein-cholesterol; MET, Metabolic equivalent; SAH, S-adenosylhomocysteine; SAM, S-adenosylmethionine*.

## Discussion

Our findings from the baseline data of the Guangzhou Nutrition and Health Cohort showed that higher serum SAH, but lower SAM and SAM/SAH were significantly associated with increased risk of SA. These associations were independent of classical cardiovascular risk factors and Hcy. The positive association of Hcy with SA risk could be mediated through SAH or SAM. Compared with SAM alone, SAM/SAH, the methylation index, had a more evident and inverse association with SA risk or CIMT levels. SAH and SAM/SAH may have stronger associations with SA than the conventional biomarker of Hcy. Future analyses utilizing follow-up data will be conducted to compare the predictive abilities of these C1 biomarkers on CVD events or other health outcomes.

This study is the first study, to the best of our knowledge conducted in an apparently healthy Chinese population to testify to the associations of core methionine metabolites with SA. Due to the insidious onset and limited tools for atherosclerosis diagnosis, as well as the poor reversibility of the disease, the identification of sensitive and valid biomarkers predicting atherosclerosis prior to its advanced stage is likely to be of particular clinical significance for the early prevention and treatment of CVD.

Studies (15, 27, 28) have shown lower SAM or SAM/SAH, and higher SAH may be more directly related to vascular damage than Hcy. SAM and SAH may be key components in the pathophysiology of the Hcy-vascular disease axis. Our results by multivariable regression analyses also showed that, after further adjustment of SAH and SAM, serum Hcy was no longer significantly associated with SA risk, and its associations with CIMT were also notably attenuated. The findings confirm that the pro-atherosclerosis effect of Hcy could be at least partly mediated by SAM or SAH.

Though Hcy is a well-established risk factor for atherosclerosis (4–6), recent randomized controlled trials did not show that reduced Hcy by folic acid and vitamin B supplementation could improve cardiovascular benefits (7–9). Devoid of alteration on SAH levels might partly explain the null effects of Hcy reduction on CVD events (10, 29). Current findings were in line with our early report in the Chinese cohort of CHD patients (13) that higher SAH was independently associated with an increased risk of cardiovascular events. Zawada et al. (15) also showed that it was SAH but not Hcy had an inverse association with SA among 420 healthy German adults. Another study among CVD patients (12) also found SAH was a more sensitive indicator than Hcy.

Homocysteine could be metabolized either *via* the transsulfuration pathway or the remethylation pathway. Within the remethylation pathway, methionine could be metabolized to SAM, which is the universal methyl group donor for a multiplicity of methylation reactions, esp. DNA methylation. SAH is a potent inhibitor of SAM-dependent transmethylation reaction. So low SAM or high SAH could result in DNA hypomethylation which leads to endothelial dysfunction, impaired cell regeneration, and atherosclerosis. *In vivo* and *in vitro* studies suggested that SAH-mediated DNA hypomethylation and associated alterations in gene expression may be a new epigenetic mechanism for the pathogenesis of atherosclerosis (30, 31).

S-adenosylhomocysteine is also a by-product of asymmetric dimethylarginine (ADMA) which is an endogenous inhibitor of nitric oxide (NO) synthase associated with an increased risk of CVD in hyperhomocysteinemia (32). SAH could impair the endothelium's capacity to regulate vascular tone by reducing the bioavailability of the vasodilator NO. A shift toward decreased NO and increased reactive oxygen species (ROS) could trigger endothelial dysfunction and promote atherogenesis. Further, in endothelial cells, SAH activated nuclear factor kappa-B (NF-κB) and induced the expression of key pro-inflammatory molecules, which also contributed to endothelial dysfunction (33).

Consistent with previous reports among CVD patients in both cross-sectional (34) and prospective studies (35), our subgroup findings also suggested that the associations of SAH or Hcy with SA risk were more evident in participants of high CVD risk profiles, and the favorable associations of SAM/SAH with SA were more notable among participants of lower CVD risk. Individuals with elevated CVD risk may be particularly susceptible to the detrimental effects of Hcy or SAH. The molecular explanations for this increased susceptibility are unclear. It might be related to accelerated oxidative stress on endothelial cells by hyper-homocysteinemia. The impaired cellular methylation may further affect the biosynthesis of a wide range of endogenous compounds, such as proteins, DNA and RNA (36).

Our findings further suggested a synergistic effect between Hcy and SAH, which were consistent with our previous reports in cohort study (13) and *in vitro* study by Lin et al. (37). SAH is the precursor of Hcy in the methionine metabolic cycle, and elevated Hcy concentrations may reversibly induce the increase in SAH. Our findings confirmed that the association between Hcy and SA might depend on the effect of SAH, and high Hcy could synergistically increase the effect of SAH on cardiovascular risk.

Our analyses found that, after adjustment of SAH and Hcy, serum SAM became significantly and inversely associated with both SA risk and CIMT level. A plausible explanation for this finding may be that a high concentration of SAM represents more methyl group substrate availability for methylation reactions, which are crucial for the repair of damaged vascular walls and the improvement of endothelial function (38). Our previous findings suggested that SAM may exert its effect under adequate SAH (16). Caudill et al. (39) fed heterozygous Cbs mice control or methyl-deficient diet for 24 weeks and found that a decrease in SAM alone was not sufficient to affect DNA methylation, whereas an increase in SAH, either alone or associated with a decrease in SAM, was most consistently associated with DNA hypomethylation. These results suggested serum SAH or SAM/SAH may be a more sensitive biomarker for cellular DNA methylation status.

Our cross-sectional findings indicated that, compared with SAM alone, SAM/SAH might be a more sensitive marker of the risk of SA. SAM/SAH is often considered the potential index of methylation and a decreased SAM/SAH often suggested a diminished cellular methylation capacity (40). SAM/SAH may be a better reflection of methylation status than SAM or SAH alone, because it contains information on both available methyl groups (conferred by the concentrations of SAM), as well as on substrate inhibition of transmethylation reactions (conferred by the concentrations of SAH). The previous study (41) also showed that alterations in the SAM/SAH may be more directly associated with vascular damage, compared with Hcy or other intermediates. By causing feedback inhibition of SAM-dependent methyltransferases, the accumulation of SAH can affect the DNA methylation pattern and lead to the promotion of chronic diseases, and the SAM/SAH is the prime regulator of the activities of the majority of methyltransferases in the cell.

Although studies have shown methionine cycle could be regulated by folic acid and vitamin B12, our analyses indicated dietary folic acid and B12 intakes had no significant association with SA, and further adjustment of folic acid and B12 levels in regression models made little alteration on the associations of methionine metabolites with SA. The possible reason could be due to the relatively large recall bias for the estimation of dietary intakes of vitamins by FFQ. It is also possible that the effect of B-vitamin might occur in people with poorer B-vitamin status. In addition, folic acid and vitamin B12 supplements promote the remethylation of Hcy to methionine in an indirect way. Future studies testing circulation or erythrocyte biomarkers for folic acid and B12 exposure are necessary.

Several limitations in our study should be acknowledged. First, given its nature as a cross-sectional design, we cannot infer the causality between C1 metabolites and SA. Second, we did not test creatinine levels despite we had excluded patients with renal failure at enrollment. Studies have shown serum creatinine was more strongly associated with SAH than Hcy (15). Future studies' inclusion of serum or urinary creatinine levels may help in results explanation. Third, dietary folic acid and vitamin B12 exposure were not confirmed by subjective biomarkers. Finally, we did not assess global DNA methylation or gene-specific changes in DNA methylation for further mechanism exploration.

## Conclusions

Our study in the Chinese middle-aged and elderly population demonstrated that higher serum SAH but lower SAM or SAM/SAH were independently associated with increased risk of SA or CIMT. SAH and SAM/SAH may be served as sensitive markers of early atherosclerosis. Future longitudinal researches are necessary to confirm whether these methionine metabolites are associated with CIMT changes and CVD incidences during follow-up.

## Data availability statement

The original contributions presented in the study are included in the article/Supplementary material, further inquiries can be directed to the corresponding author/s.

## Ethics statement

The studies involving human participants were reviewed and approved by Institute Ethics Committee of Sun Yat-sen University. Written informed consent to participate in this study was provided by the participants' legal guardian/next of kin.

## Author contributions

JX wrote this manuscript, detected the C1 metabolites of the methionine cycle, and had the primary responsibility for the final content. YY analyzed the data and detected the C1 metabolites of the methionine cycle. XC and YT participated in the collection of biological samples and data. WL developed the overall research plan, conducted research supervision, and reviewed this manuscript with ZL. YC provided research guidance and reviewed this manuscript. QL revised the manuscript. The manuscript is an original work and the final version has been read and approved by all authors.

## Funding

This study was jointly supported by the National Natural Science Foundation of China (Grant No. 81973022).

## Conflict of interest

The authors declare that the research was conducted in the absence of any commercial or financial relationships that could be construed as a potential conflict of interest.

## Publisher's note

All claims expressed in this article are solely those of the authors and do not necessarily represent those of their affiliated organizations, or those of the publisher, the editors and the reviewers. Any product that may be evaluated in this article, or claim that may be made by its manufacturer, is not guaranteed or endorsed by the publisher.
