# Supplementary material for: Higher S-adenosylhomocysteine and lower ratio of S-adenosylmethionine to S-adenosylhomocysteine were more closely associated with increased risk of subclinical atherosclerosis than homocysteine
Source: Front Nutr. 2022 Aug 10;9:918698. doi: 10.3389/fnut.2022.918698 (PMC9399787; doi:10.3389/fnut.2022.918698)
Supplement: Supplementary file 1 [file Data_Sheet_1.docx]

**Table S1.** HPLC-MS operating conditions

1. Summary of MS settings

| Compound | Q1 (*m/z*) | Q3 (*m/z*) | Dwell time (ms) | Fragmentor (V) | CE (V) |
| --- | --- | --- | --- | --- | --- |
| SAM | 399.1 | 250.1 | 80 | 105 | 13 |
| SAH | 385.1 | 136 | 80 | 110 | 21 |
| Homocysteine | 136.1 | 90.1 | 80 | 75 | 7 |
| SAM-D3 | 402.1 | 250 | 80 | 110 | 17 |
| SAH-D4 | 389.1 | 136 | 80 | 105 | 21 |
| Homocysteine-D4 | 140 | 94 | 80 | 75 | 11 |

Abbreviations: SAH: S-adenosylhomocysteine; SAM: S-adenosylmethionine.

**B.** Summary of HPLC settings

| Column temp. | 35℃ |  |  |
| --- | --- | --- | --- |
| Flow rate | 0.15 mL/min |  |  |
| Mobile phase | A- Purified water + 1% formic acid |  |  |
|  | B- Methanol + 1% formic acid |  |  |
| Gradient | Time (min) | % A | % B |
|  | 0 | 97 | 3 |
|  | 2 | 95 | 5 |
|  | 4 | 90 | 10 |
|  | 9 | 50 | 20 |
|  | 11 | 0 | 100 |
|  | 13 | 0 | 100 |


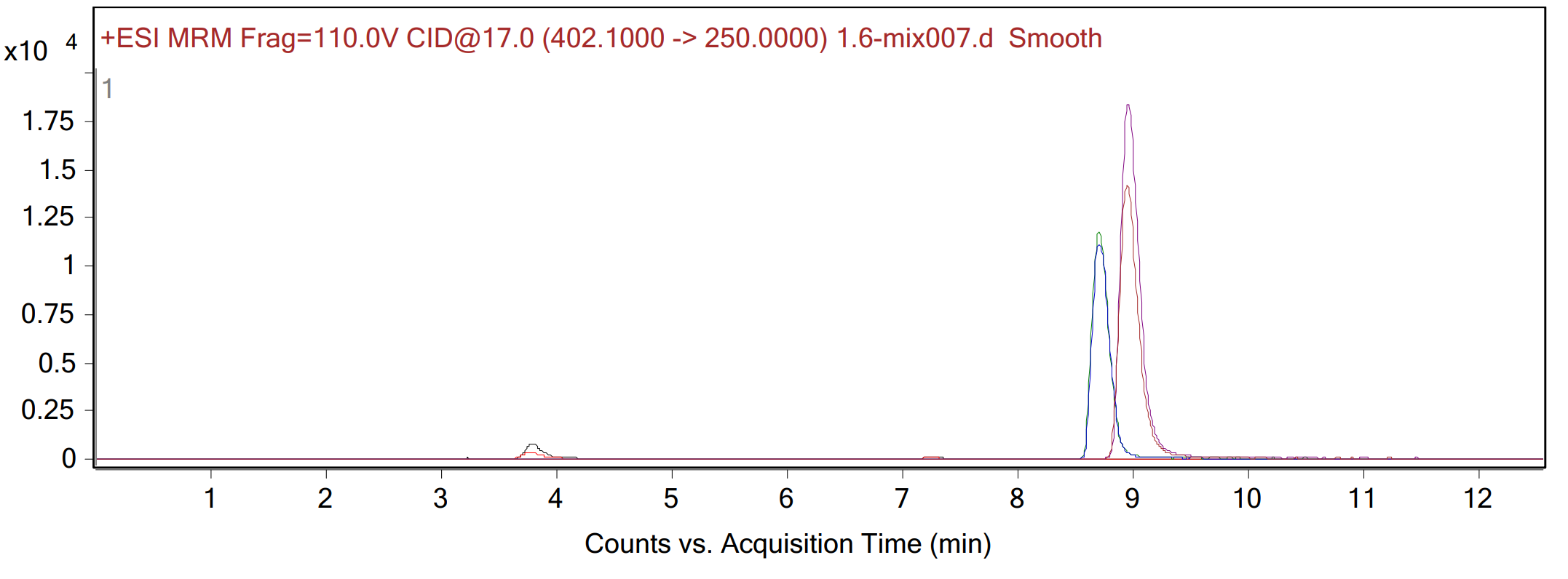


Homocysteine

SAH

SAH-D4

Homocysteine-D4

SAM-D3

SAM

**Fig S1.** Typical chromatograms of C1 metabolites of the methionine cycle

Abbreviations: SAH: S-adenosylhomocysteine; SAM: S-adenosylmethionine.

**Table S2.** Linearity of calibration.

| Compound | Linear equation | R^2^ | Linear range | LOD (nM) | LOQ (nM) |
| --- | --- | --- | --- | --- | --- |
| SAM | Y=0.6653X+1.6914*10^-4^ | 0.9999 | 2.5-400 nM | 0.1 | 0.05 |
| SAH | Y=0.5070X+6.5910*10^-4^ | 0.9999 | 2.5-400 nM | 0.5 | 0.24 |
| Homocysteine | Y=2098.2591X+1.6914*10^-4^ | 0.9974 | 0.0625-10 µM | 14 | 7 |

Abbreviations: LOD: Limit of detection; LOQ: Limit of quantification; SAH: S-adenosylhomocysteine; SAM: S-adenosylmethionine.

**Table S3.** Recovery and precision at three different concentration levels (*n*=6 per day, per level)

| Analyte | Target concentration | Recovery (%) | Intra-day precision (%) | Inter-day precision (%) |
| --- | --- | --- | --- | --- |
| SAM | 5.0 nM | 97.9 ± 2.6 | 2.9 | 3.6 |
|  | 20 nM | 100.4 ± 2.9 | 2.9 | 2.0 |
|  | 100 nM | 99.1 ± 0.8 | 0.5 | 0.4 |
| SAH | 5.0 nM | 104.8 ± 2.5 | 5.4 | 6.9 |
|  | 20 nM | 103.3 ± 1.2 | 2.6 | 1.3 |
|  | 100 nM | 100.5 ± 2.1 | 2.2 | 0.9 |
| Homocysteine | 0.125 μM | 103.7 ± 3.4 | 5.2 | 1.9 |
|  | 0.5 μM | 105.6 ± 1.0 | 2.0 | 4.1 |
|  | 5.0 μM | 102.3 ± 0.7 | 2.7 | 0.9 |

Abbreviations: SAH: S-adenosylhomocysteine; SAM: S-adenosylmethionine.

**Table S4.** *P* values of interaction for the risk of subclinical atherosclerosis between quartiles of serum C1 metabolites of the methionine cycle and selected cardiovascular risk factors, Guangzhou Nutrition and Health Cohort (*n*=2991)^1^

| *P* for interaction between metabolites and Selected CVD risk factors | SAM | SAH | Homocysteine | SAM/SAH |
| --- | --- | --- | --- | --- |
| Age | 0.548 | 0.883 | 0.020 | 0.874 |
| Sex | 0.156 | 0.639 | 0.163 | 0.134 |
| BMI | 0.802 | 0.601 | 0.948 | 0.719 |
| Smoking | 0.598 | 0.768 | 0.912 | 0.558 |
| Alcohol drinking | 0.304 | 0.109 | 0.149 | 0.239 |
| Folic acid intake | 0.839 | 0.241 | 0.366 | 0.187 |
| Vitamin B12 intake | 0.914 | 0.607 | 0.314 | 0.540 |
| LDL-c | 0.069 | 0.717 | 0.334 | 0.029 |
| HDL-c | 0.090 | 0.737 | 0.593 | 0.778 |
| Total triglyceride | 0.846 | 0.510 | 0.622 | 0.588 |
| Fasting glucose | 0.397 | 0.151 | 0.819 | 0.016 |
| Homocysteine | 0.204 | 0.001 | - | 0.010 |

1. Interaction analyses were conducted by inclusion of a product term of categorized cardiovascular risk factor with quartiles of C1 metabolites (SAM, SAH, homocysteine and SAM/SAH) in the multivariable logistic regression model by enter methods. The adjusted covariates included age (years), sex (men or women), BMI (kg/m^2^), waist circumference (cm), education (≤6, 7-12, or >12 years), monthly income (<1500, 1500–3000, or >3000 yuan), physical activity (MET•h/d), smoking status (yes or no), alcohol drinking (yes or no), tea drinking (yes or no), systolic blood pressure (mmHg), energy intake (kcal/d), fat intake (g/d), protein intake(g/d), dietary folic acid and vitamin B12 intake (µg/d), LDL-c (mmol/l), HDL-c (mmol/l), total triglyceride (mmol/l), fasting glucose (mmol/l) and homocysteine (μmol/l). Subsequent subgroup analyses (see **Table 5**) were only performed for variables with *P* for interaction less than 0.15.
2. Abbreviations: BMI: Body mass index; HDL-c: High-density lipoprotein-cholesterol; LDL-c: Low-density lipoprotein-cholesterol; MET: Metabolic equivalent; SAH: S-adenosylhomocysteine; SAM: S-adenosylmethionine.
